# Valedictory Address of M. H. O’Daniel, of Bullard’s, Ga.

**Published:** 1881-03

**Authors:** 


### VALEDICTORY ADDRESS ON THE PART OF THE GRADUATING CLASS IN ATLANTA MEDICAL COLLEGE, DELIVERED AT THE ANNUAL COMMENCEMENT, MARCH 2, 1881, BY M. H. O'DANIEL, OF BULLARD'S, GA.

---

LADIES AND GENTLEMEN :

It is with both reluctance and delight that I rise to address you to-night. With reluctance, because I fear my ability to do justice to this august occasion. With pleasure, because it brings up pleasant memories, spreading them in their rich and glowing colors before the ardent soul of youth, and binding them about the heart of man with the golden, bright, and silken band of this blessed, smiling hour.

As I look round about me this evening, I can but gaze with astonishment, and am even lost in wonder when I behold this crowded hall in such silent expectation. What means this eager, anxious throng? What means these proud and manly forms? These fair and smiling faces? What means each close attentive ear? Your steady, gazing eyes?

Me thinks I hear, in accents clear, from this fair audience to-night, "We come to witness the laying of the corner stone of your future weal or woe."

Throw back the curtain of by-gone ages and gaze for one moment upon the dark and groveling state of man. Go into the remotest part, at the founding of Babylon and Tyre. Contemplate, if you please, the wretchedness of their days, and the foul, muddy cast of their most heart-rending deeds. Visit Greece and Rome, and there, too, ignorance will stare you in the face, superstition make you shudder, your spirit would tremble and quail and your heart grow sick and faint to view the spot where crimes of midnight darkness strangled smiling innocence upon the downy bed of forgetfulness and sweet repose.

There, where laws are violated and abused, where conscience is forgotten and lost, where the guilty go unpunished and the innocent suffer the pangs of death· Where the blood of the son and sire mingled and flowed together, or where the weary soul of man had taken its leave, had flown.

Where smiling babes were snatched from their mother's breast. Where torch and sword played havoc with home and soul.

Where me thinks hell did gape, earth tremble, and heaven knit her clouded brow mid nightly peals of thundering wrath.

But the order of the world is changed. We live in a new era, and enlightenment stamps the hours with improvement as they pass swiftly by.

Superstition has been strangled, ignorance expelled, bloody crime checked in its wild career, while peace and order now do flourish upon our fair and blooming earth. No more graven images, no more sacrificing human life to appease the anger of the weather gods. No more throwing babes into the hungry waves to attone for the crimes of the blood-thirsty and the damned. The false gods are broken and drowned, their worshippers have passed away.

Now, let these idols be forgotten, while morals and religion hold the sway with heaven's propitious smiles. Their faries have vanished into air, their graces mouldered in decay, while truth and right and honors fair now deck the brow of the smiling day.

Now, midst the general advancement of the ages, in cultivation of heart and health, in the progress of arts and sciences, of painting and poetry, of music and love, where have there been greater attainments than in this, our own blessed land of America?

Where has there been sweeter oratory than from the lips of our distinguished divines, Beecher, Talmage, Brantly, Haygood, Hall and Mell? Where has more thrilling eloquence stirred the soul of man than from the musical voices of Stephens, Toombs and Hill? And last, but not least, I would ask where stands the medical profession, today? Me thinks I hear in proud, stentorian tones, the happy tidings, reverberated from hill top to hill top, from valley to valley, from the snow capped sumit of chilly Main down to our own sunny land of magnolias and bays. Where is genius brighter than here? Where is learning more profoundly deep? Where has the medical gale blown more propituously, than in our own fair land? and the same mountains catch up the sound and the hills and valleys again resound. We are inferior to none on this beautiful sphere, but will peer with any under the blue vault of heaven, and now with our shoulders still to the wheel, our hearts to the task, our brain to action, we shall carry on the good work, curing diseases, relieving pain and shall be an honor to our noble profession, and a blessing to humanity, and a glory to God.

And now, kind people of Atlanta, and friends, allow me, in behalf of my class, to thank you for your presence, and attention this evening, and also for the general hospitality shown us during our sojourn in your city.

And now, to you, most noble and honored trustees of Atlanta Medical College, I turn my attention to you for a while, and will endeavor, though in a feeble way, to express our gratitude to you for your kindness, and our highest appreciation of your incalculable worth, in behalf of this our beloved *alma mater*.

We feel that our college is growing, and her influence is being felt and appreciated, at home and abroad. Affected by your wise administration, by your kindly dispensation of justice, your morals and religion, your honor and your learning.

You have studied our interest, you have advocated our cause, you have maintained our rights and privileges, and blessed us in our own noble persuit.

You have been honest in purpose, zealous in labor and faithful to perform every obligation. We know that we have been in safe keeping, and do feel secure under your vigilant care. We love you because you do your duty, and honor you because we love you so.

The Atlanta Medical College stands among the first of the land, and it is in part to you that she owes her greatness to-day, for indeed is she proud of her name, and well can she glory in her worthy claims. She is blessed with facilities, rewarded with intelligence, surrounded by wealth at the capital of Georgia, amid civilization and learning, refinement and integrity. Encouraged by all this, stimulated by the rapidly progressing age, she can't stand still; she must move on, and success is inevitable, while honor lingers in her classic halls and glory lays the laurel wreaths upon her fair and placid brow. Constantly is she tossing her proud head to the heavens and throwing her arms farther to the South, bidding Southern students to fly to her bosom, and welcome in her fond embrace, and then to the North does she turn in triumph and bid them come and join the band. She beckons them from the east, from the west, and from across the mighty deep. They learn her matchless name, and come and slake their thirst at her refreshing fountain.

Time bids me hasten on, and I must leave you for a season, for I cannot dwell upon the things I love so well, but one word more before I say adieu, noble gentlemen, honored and beloved trustees.

Remember, you shall have the prayers of this grateful class. No matter how distantly separated we may be.

Sweet memories of you will rise up like pleasant dreams, and flit before our minds, and we shall catch them up, press them to our souls, and be loth to let them go. So, kind friends, I say farewell.

Here sit our learned and beloved faculty. Ever courteous and true, with honest hearts beating in unison with duty, and wills to do the right, who spurn wrong, who are ever willing to dispense impartial justice and extend mercy.

Gentlemen, we welcome you here this evening, and our souls do rejoice to behold your pleasant countenances, as we remember your perseverance, your earnestness, your faithful and continuous instruction and impressive lectures.

You have not for once abated your untiring efforts in our advancement, and constantly have you had in view our successful development. Medicine can but flourish at this, your noble shrine of learning, and clap her hands and be glad, oh, thou sweet and noble benefactor! thou life-restorer! thou God-sent blessings upon poor, suffering humanity! It is with joy and delight that we hail your glory, and with Heaven's high pride, that we elevate your banner. We are proud that we are Americans in a God-favored spot of Georgia, where man is free to act and think and medicine not restricted.. Thank God, we shall plant thy roots still deeper in a family soil, that is even conducive to thy honored growth, and rejoicing mid smiling at thy benignant sway. Thou shalt spring up around, as it were, and stretch forth thy branches to the winds of the earth, and thy heads shall tower in the skies and smile. Sickness and sorrow shall vanish at thy approach, like the hare before the eagle. Pain and death shall take wings and fly, and health and comfort rule, where once did misery.

Fellow-Comrades: We have been wont to contemplate this period of our lives with gayety of spirits and musings to enjoy the sweet-breathed consolation in the freedom of our after years. Time to come, clad in the rich robe of her beauty, spreads out her gorgeous colors to the exquisite imaginings of our hopeless brain to make the scene a garden spot, and our land a land of dreams, whose golden moments rejoice with the soul in glad delight, but leave no stain nor blotch of their harmless revel. No ghost, nor demon, nor foul spirit damned, darkens with the shadow of his sepulchral wing the sunlit region over which our proud thoughts have triumphantly flown. No grievous sound, no midnight wail breaks in rudeness upon fancy's ear to tinge our buoyant souls with gloom. No clouds in the bosom of our smiling skies threatening storm and flood; but hope, the bright-winged messenger of heaven, gay of plumage and sweet of song, like the bird of paradise pours forth its strains of unpremeditated art, and with its gentle melodies lulls every fear to rest.

I can see in every eye the flaming hopes of success; I can read in every heart the resolution to perform his duty; I can mark out as it were the resplendant bands, that encircle your souls, and even see them smile as the golden clasps of Heaven. But let us not dream all the days of our lives; these are mere speculations, sweet fancies glittering before youth's happy brain, and now stern reality must take its course, and the winds blow wherein they list. Remember, dear friends, we have assumed a profession of the gravest responsibilities; and though our labors may be great, though disappointments may befall us, thick and fast; though our sufferings make us shiver, and sorrow bow us down; yet let us like men with cheerful faces and willing hearts, go nobly to our honored tasks. Let us all be lovers of learning and searches of science, and our labors will be lessened, and our pleasures the more increased. Let us not turn a deaf ear to the cries of our afflicted people; stand not with your hands folded and your eyes and senses closed to that grim monster—death. Strike your brain and gird about you the armor of your learning. What! Hear ye not the wail of that helpless child? Hear ye not the moan of that frail and agonizing mother? See ye not the sweat drops of death trickle down that pallid brow? Can we stand still while death with the sickle in his hand is fairly sweeping from this earth of ours into eternity, poor, faint and suffering mortals? Can we see the bones all broken and mangled? Can we see the flesh torn and the blood streaming, and the human frame agonizing, and writhing in all unearthly pain, and withhold the healing art? Can we stand untouched, and unmoved like a rock? No! a thousand times no! Let the hills and the valleys catch up the sound, and echo no! Reason holds up her hands against it; our profession disdains it; humanity looks down with scorn, and we shall be held accountable by that just God, the last tribunal for the same. It is true many times we shall feel faint hearted and despondent; great mountains like mighty monsters shall rise up in their midnight garb and foam, and make us tremble!

> "The deep toned thunder shall roll across the vaulted sky,
> And the red-forked lightning flash its fires from on high."

The raging storms shall howl around; the rains of life shall patter down, while the winds from off the winter snow shall make you chill and dreary.

Your skies will not always be bright and sunny, nor your lives made up with smiles. Earth at times shall lose its charms, and the Heavens seem made of brass; the blushing rose shall hide its face, and the dark and lowering clouds shall hover about your spirits, and gloom overshadow your now fair brow, and the billows of life shall roll dark and deep, and the dashing waves surge and sweep; the winds of adversity may blow upon you, and sorrow's breath, like a vagrant tramp, shall steal about your homes, and leave a damper there; but be not discouraged "for in every one's life some rain must fall, some days be dark and dreary."

Buckle on the armor of faith, and let duty be your watchword; stamp upon your brows the ornament of truth; impress upon your hearts fidelity and right, and your care, for 'tis duty that must govern our actions here below, and must be the luminary that lights our spirits home. Let reason shine within the realms of your understanding.

Gird about your loins the preparations for eternity, and let love be without dissimulation; deep and long at the fountain of wisdom, and bow in reverence at the shrine of God, and remember that life's gay attire must soon be surrendered for the grave's pale shrowd, and the freedom of earth for the confinement of the coffin and the tomb.

So let us be true to ourselves, our country and our God, and let Heaven be our aim, our aspiration, our hope, and then as down the stream of time in our frail bark we glide, we can wake, listen and welcome the music, as it falls in mournful strains upon the ear.

